# The diagnostic performance of dual-energy CT imaging in cervical lymph node metastasis of papillary thyroid cancer: a meta-analysis

**DOI:** 10.3389/fmed.2024.1457307

**Published:** 2024-11-06

**Authors:** Aiping Han, Yanqiu Liu, Lingxian Cai, Jing Xie, Shouzhang Hu

**Affiliations:** ^1^Department of Radiology, Affiliated Geriatric Hospital of Wuhan University of Science and Technology, Wuhan, China; ^2^School of Public Health, Department of Medical, Wuhan University of Science and Technology, Wuhan, China; ^3^Department Hospital, Wuhan University of Science and Technology, Wuhan, China

**Keywords:** dual-energy CT, cervical lymph node metastasis, papillary thyroid cancer, meta-analysis, diagnostic efficacy

## Abstract

**Purpose:**

This meta-analysis aimed to evaluate the diagnostic efficacy of dual-energy computed tomography (DECT) in detecting cervical lymph node metastasis among papillary thyroid cancer (PTC) patients.

**Methods:**

A comprehensive search across PubMed, Embase, and Web of Science databases was conducted to identify pertinent publications up to May 2024. This search focused on studies examining the diagnostic accuracy of DECT in detecting cervical lymph node metastases in PTC patients. We employed a bivariate random-effects model to calculate pooled sensitivity and specificity of DECT. The degree of heterogeneity in the studies was quantified using the *I*^2^ statistic. Furthermore, the Quality Assessment of Diagnostic Accuracy Studies-2 (QUADAS-2) tool was utilized to evaluate the methodological quality of the included studies.

**Results:**

This meta-analysis encompassed 14 articles, collectively involving 1,615 patients. The pooled sensitivity, specificity, and AUC for DECT in detecting cervical lymph node metastases in PTC patients were 0.81 (95% CI: 0.76–0.85), 0.86 (95% CI: 0.80–0.91), and 0.89 (95% CI: 0.86–0.92), respectively. According to Fagan’s nomogram, for DECT, with a pre-test probability of 50%, the post-test probability was calculated as 85% for a positive result and 18% for a negative result. Deeks’ funnel plot asymmetry test showed no significant publication bias was observed for DECT (*p* = 0.28).

**Conclusion:**

Our meta-analysis indicates that DECT demonstrates superior sensitivity and specificity in cervical lymph node metastasis among PTC patients. To corroborate these findings and evaluate their clinical applicability, further prospective studies are necessary.

## Introduction

Cervical lymph node metastasis in papillary thyroid cancer (PTC) is a critical factor for risk stratification and treatment planning (1–3). PTC is the predominant type of thyroid cancer, representing 80–88% of cases (4). Although PTC generally exhibits a slow progression and favorable prognosis, aggressive behaviors and recurrence, including extrathyroidal extension (ETE) and cervical lymph node metastasis, are not uncommon (5–7). The occurrence of cervical lymph node metastasis, which can be present in 30–80% of PTC patients (8), often complicates early detection due to the indistinct characteristics of thyroid cancer nodules (9). Therefore, accurate preoperative diagnosis of cervical lymph node metastasis is clinically relevant for optimizing treatment planning and improving patient prognosis.

Traditionally, the assessment of cervical lymph node metastasis in PTC involves ultrasound (US), computed tomography (CT), and fine-needle aspiration biopsy (FNAB). While US is the primary screening tool, its sensitivity is limited (10), particularly in identifying diminutive lymph nodes or those situated at levels VI and VII (11), Moreover, US’s diagnostic accuracy is significantly influenced by the operator’s experience (12). Conventional CT, though useful in providing anatomic details, often falls short in distinguishing benign from malignant lymph nodes based solely on size and morphology (13). FNAB, while definitive, is invasive and subject to sampling errors (14). These limitations necessitate the exploration of advanced imaging modalities to enhance diagnostic accuracy.

DECT, an advanced imaging technique, offers a promising solution to the limitations of traditional CT methods. DECT employs a rapid alternation between high-energy (140 kVp) and low-energy (80 kVp) datasets during a single gantry rotation (15). This approach generates a spectrum of monochromatic images and facilitates material decomposition (16). DECT not only provides enhanced imaging capabilities but also yields additional quantitative data, including iodine concentration and effective atomic numbers. Such detailed information significantly improves the differentiation between benign and malignant tissues (17–20). Despite its potential, the use of DECT in detecting cervical lymph node metastasis in PTC is not yet universally accepted, with some studies reporting conflicting results regarding its diagnostic performance (21, 22). This controversy highlights the need for a comprehensive analysis of existing literature to elucidate the true diagnostic performance of DECT in this context.

Therefore, this meta-analysis aims to systematically review and synthesize current evidence on the diagnostic performance of DECT imaging in cervical lymph node metastasis of PTC.

## Methods

The meta-analysis was meticulously conducted in full compliance with the Preferred Reporting Items for Systematic Reviews and Meta-Analyses of Diagnostic Test Accuracy (PRISMA-DTA) guidelines (23). Furthermore, the protocol for this study is registered with PROSPERO (CRD42023480032).

### Search strategy

A comprehensive search across PubMed, Embase, and Web of Science databases was conducted to identify pertinent publications up to May 2024. This search utilized specific key terms: ‘Dual-energy computed tomography,’ ‘Thyroid neoplasms,’ and ‘Lymph node metastasis.’ Further details can be found in Supplementary Table S1. Furthermore, reference lists of the included studies were manually reviewed to identify additional relevant articles.

### Inclusion and exclusion criteria

Studies were selected for this meta-analysis based on the following PICOS criteria: Population (P): patients diagnosed with PTC in a preoperative condition; Intervention (I): DECT for diagnostic evaluation; Comparison (C): not applicable; Outcomes (O): sensitivity and specificity of DECT in diagnosing cervical lymph node metastasis; Study Design (S): retrospective or prospective studies.

Excluded from consideration were (1) duplicate articles, (2) abstracts without full texts, editorial comments, letters, case reports, reviews, meta-analyses, (3) publications with irrelevant titles or abstracts, and (4) non-English full-text articles. Additionally, studies with incomplete or ambiguous data critical for assessing the sensitivity or specificity of the imaging modality under investigation were omitted.

### Quality assessment

Two researchers independently assessed the quality of the included studies using the Quality Assessment of Diagnostic Accuracy Studies (QUADAS-2) tool. This instrument evaluates four key domains: (1) patient selection; (2) the index test; (3) the reference standard; and (4) flow and timing, focusing on the risk of bias. Additionally, the first three domains are examined for concerns regarding applicability. Each study was reviewed and analyzed by at least two authors. In cases of disagreement, resolutions were achieved through consensus or by involving a third reviewer. The methodological quality of the studies was assessed using RevMan software (Version 5.4).

### Data extraction

Data extraction from the included articles was independently performed by two researchers. This process involved collecting various details, including author names, publication year, study characteristics (country, study design, analysis period, and reference standard), patient demographics (number of patients, number of lesions, mean age, gender distribution, and prior conventional tests), and technical parameters (scanner modality, tube voltage and current, reconstructed slice thickness pitch, contrast agent dosage, and analysis method). In instances of disagreement, the researchers engaged in discussion to reach a consensus, thereby ensuring accuracy in the extracted data.

### Statistical analysis

Utilizing a bivariate random-effects model, we reported the pooled sensitivity and specificity of DECT as estimates, each with 95% confidence intervals (CIs). The summary receiver operating characteristic (SROC) model was employed to generate the SROC curve and the AUC. A statistically significant difference in performance between the two modalities was inferred if their 95% confidence intervals did not overlap.

Heterogeneity among pooled studies was assessed using the *I*^2^ statistic. In cases where substantial heterogeneity was detected (*I*^2^ ≥ 50%) for DECT, a meta-regression analysis was performed to identify potential sources of this heterogeneity. To assess publication bias, Deeks’ funnel plot asymmetry test was employed. All analyses were conducted with Stata 15.1. Statistical significance was defined as a *p* value less than 0.05.

## Results

### Study selection

**Figure 1:**
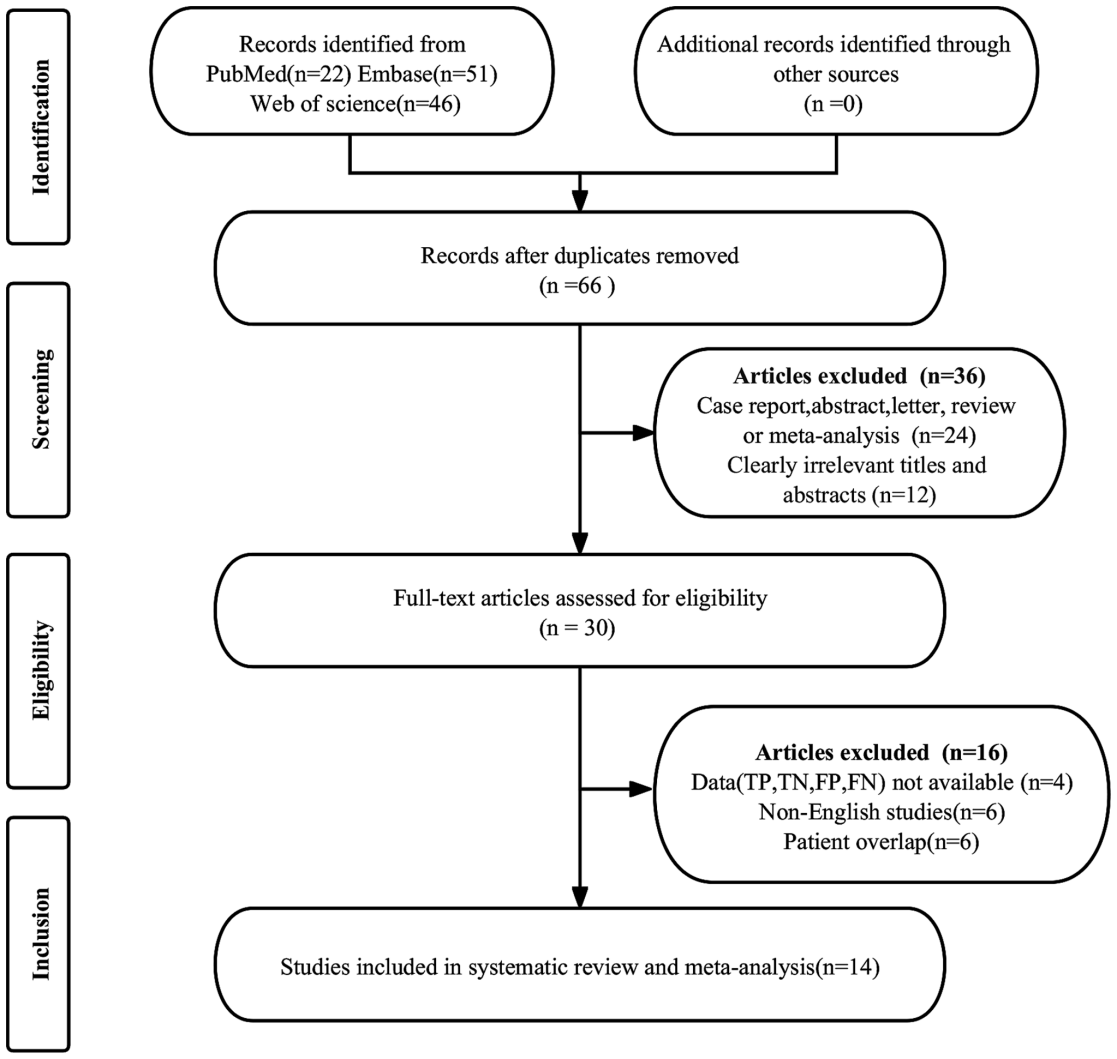
PRISMA flow diagram illustrating the study selection process.

The initial search produced 119 publications. Of these, 53 were duplicates and were thus removed. An additional 36 articles were excluded for not meeting the eligibility criteria. Subsequent in-depth examination of the remaining 30 full-text articles led to the further exclusion of 16 studies. Reasons for exclusion included the unavailability of necessary data (true positive, false positive, false negative, and true negative) in 4 studies, overlapping patient cohorts in 6 studies, and non-English language in 6 studies. Ultimately, 14 articles assessing the diagnostic efficacy of DECT were included in the meta-analysis (11, 21, 22, 24–34). The process of article selection is delineated in Figure 1, adhering to the PRISMA flow diagram format.

### Study description and quality assessment

The 14 selected studies encompassed a total of 1,615 patients with PTC, with individual study sample sizes ranging from 31 to 406. Among these studies, nine were retrospective and five were prospective. Analytically, 5 articles employed a patient-based approach, and 9 utilized a lesion-based analysis. As for reference standards, 12 articles used pathology, and 2 combined pathology with follow-up imaging. In terms of diagnostic techniques, 7 articles adopted quantitative analysis, 3 relied on visual analysis, and 4 integrated both quantitative and visual methods. Table 1 summarizes the study and patient characteristics specific to DECT, while Table 2 details the technical aspects of the studies.

**Table 1:** Study and patient characteristics of the included studies.

Author	Year	Study characteristics						Patient characteristics			
		Country	Study design	Analysis	Period	Reference standard	Number of patients	Number of lesions	Mean age ± SD	Male/Female	Previous conventional tests
Huang et al.	2020	China	Retro	PB	From 2013 to 2018	Pathology	95	NA	43.32 ± 11.34	24/71	US
Jin et al.	2022	China	Retro	LB	From 2018 to 2020	Pathology	78	293	40.75 ± 12.62	22/56	US
He et al.	2019	China	Pro	LB	From 2016 to 2017	Pathology	51	212	NA	16/35	NA
Li et al.	2019	China	Pro	LB	From 2017 to 2017	Pathology	30	99	41.6 ± 14.8	13/17	US
Liu et al.	2015	China	Pro	LB	From 2012 to 2013	Pathology	45	175	34.05 ± 7.81	11/34	US
Ren et al.	2023	China	Retro	PB	From 2021 to 2022	Pathology	348	NA	47.06 ± 11.52	79/269	US
Wu et al.	2021	China	Retro	LB	From 2018 to 2019	Pathology and follow-up imaging	35	206	39.79 ± 13.58	6/29	US
Zhao et al.	2023	China	Retro	PB	From 2016 to 2021	Pathology	140	NA	44.29 ± 13.95	19/121	US
Yoon et al.	2021	South Korea	Pro	PB	From 2019 to 2020	Pathology	102	NA	46 ± 15	32/70	US
Zhou et al.	2020	China	Retro	LB	From 2016 to 2019	Pathology	108	177	38.6 ± 13.2	49/59	NA
Zhou et al.	2023	China	Retro	LB	From 2021 to 2022	Pathology	92	273	41	23/69	NA
Zhou et al.	2022	China	Pro	LB	From 2020 to 2021	Pathology	54	157	33	13/41	NA
Li et al.	2017	China	Retro	LB	From 2016 to 2016	Pathology	31	115	39.4	9/22	US
Zou et al.	2021	China	Retro	PB	From 2016 to 2019	Pathology and follow-up imaging	406	NA	46.87 ± 12.85	84/322	US

Pro, prospective; Retro, retrospective; PB, patient-based; LB, lesion-based; US, ultrasound; NA, not available.

**Table 2:** Technical aspects of included studies.

Author	Year	Scanner Modality (System)	Tube voltage and current	Reconstructed slice thickness (mm)	Pitch	Dose of contrast agent	Analysis method	TP	FP	FN	TN
Huang et al.	2020	Somatom Defniton Flash; Siemens Healthcare	Tube voltage 80/Sn 140 kVp; 150 mAs and automatic tube current	0.75	0.7	65 mL	Quantitative	43	7	16	29
Jin et al.	2022	Discovery CT750 HD scanner; GE Healthcare	Tube voltage 80/140 kVp; tube current 360 mA	1.25	0.984	1.6 mL/kg	Visual and Quantitative	106	45	11	131
He et al.	2019	Somatom Force; Siemens Healthcare	Tube voltage 80/150 kVp; tube current 130 mAs and 65 mAs	1.5	0.6	85 mL	Quantitative	112	3	12	85
Li et al.	2019	CT750 HD Scanner; GE Healthcare	Tube voltage 80/Sn 140 kVp; Tube current 260 mA	5	0.984	90 mL	Quantitative	61	2	9	27
Liu et al.	2015	Discovery CT750 HD scanner; GE Healthcare	Tube voltage 70-keV; Tube current 550 mA	1.25	1.375	NA	Quantitative	46	13	17	99
Ren et al.	2023	Somatom Force; Siemens Healthcare	Tube voltage 80/Sn 150 kVp; Tube current 118 mAs and 59 mAs	1	0.6	80 mL	Visual	139	78	40	91
Wu et al.	2021	Discovery CT750 HD scanner; GE Healthcare	Tube voltage 80/140 kVp; Tube current 260 mA	1.25	0.984	1.2 mL/kg	Visual and Quantitative	70	33	10	93
Zhao et al.	2023	Somatom Defniton Flash; Siemens Healthcare	Tube voltage 80 kV/Sn140 kV;	1	NA	1.0 mL/kg	Quantitative	62	10	26	42
Yoon et al.	2021	Somatom Force; Siemens Healthcare	Tube voltage 80/140 Sn kVp	3	0.6	90 mL	Visual and Quantitative	38	5	15	46
Zhou et al.	2020	Somatom Force; Siemens Healthcare	Tube voltage 80/ Sn 150 kVp; Tube current, 118 mAs and 59 mAs	1.5	0.7	75 mL	Visual	64	6	12	96
Zhou et al.	2023	IQon Spectral CT, Philips Healthcare	Tube voltage 120 kVp; Automatic tube current	1	1.1	75 mL	Visual and Quantitative	107	33	13	120
Zhou et al.	2022	IQon spectral CT, Philips Healthcare	Tube voltage 120 kVp; Automatic tube current	1	1.1	75 mL	Quantitative	49	7	27	74
Li et al.	2017	Discovery CT750 HD scanner; GE Healthcare	Tube voltage 80/140 kVp; Tube current, 260 mA	1.25	0.984	90 mL	Visual	67	2	16	30
Zou et al.	2021	Somatom Defniton Flash; Siemens Healthcare	Tube current 600 mA	1	0.9	1 mL/kg	Quantitative	93	39	35	239

TP, true positive; FP, false positive; FN, false negative; TN, true negative.

**Figure 2:**
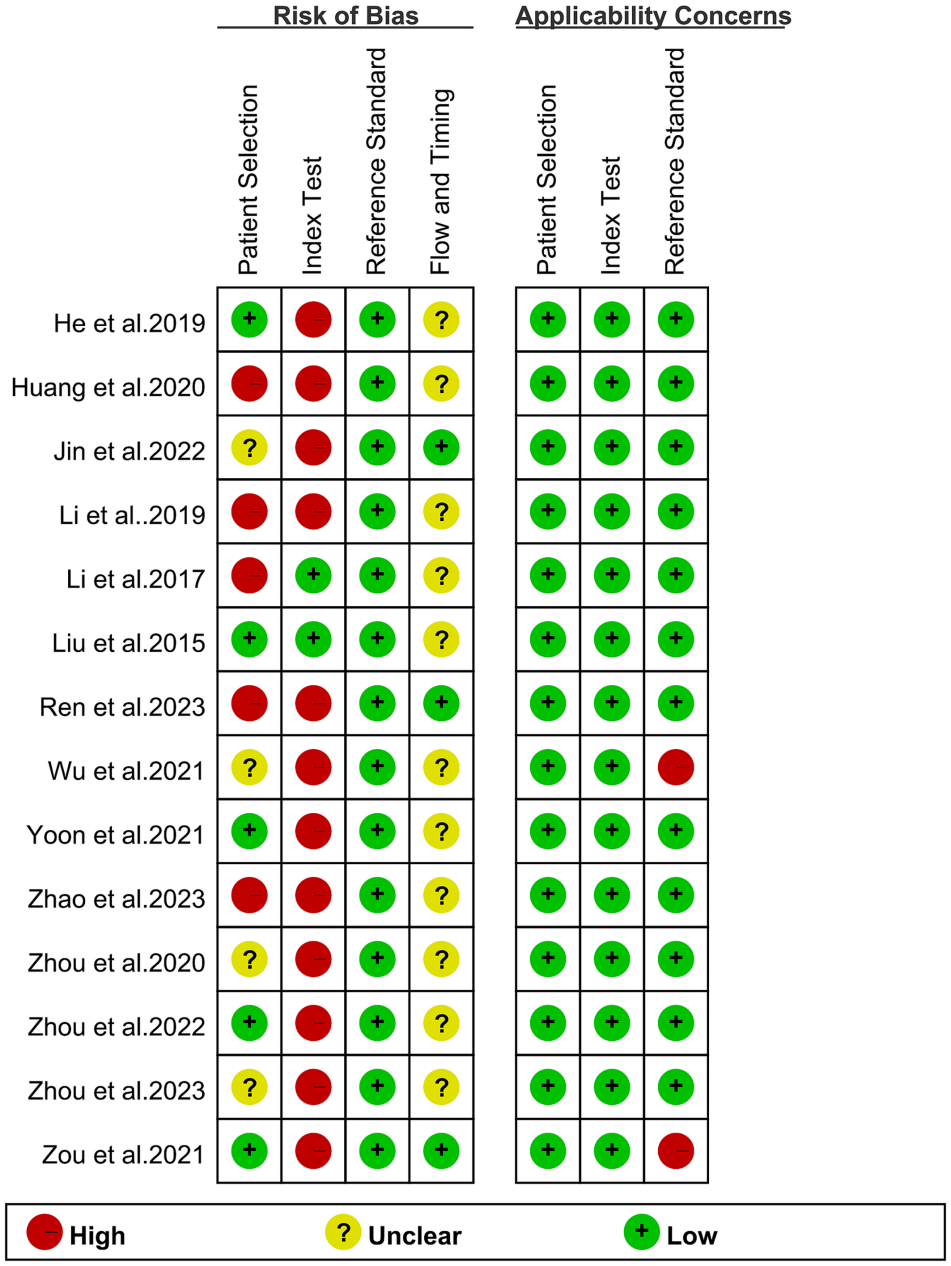
Risk of bias and applicability concerns of the included studies using the Quality Assessment of Diagnostic Performance Studies QUADAS-2 tool.

The assessment of bias for each study, conducted using the QUADAS-2 tool, is illustrated in Figure 2. With respect to patient selection, five studies were deemed “high risk” due to non-standard exclusions. In the context of the index test, 12 studies fell into the “high risk” category because of employing non-predefined cut-off values. Concerning the reference standard, two studies were categorized as “high risk” owing to reliance on clinical imaging follow-up in the original investigations. Despite these particular points of concern, the evaluation affirmed the overall quality of the included studies.

### Diagnostic performance of DECT in cervical lymph node metastasis of PTC

**Figure 3:**
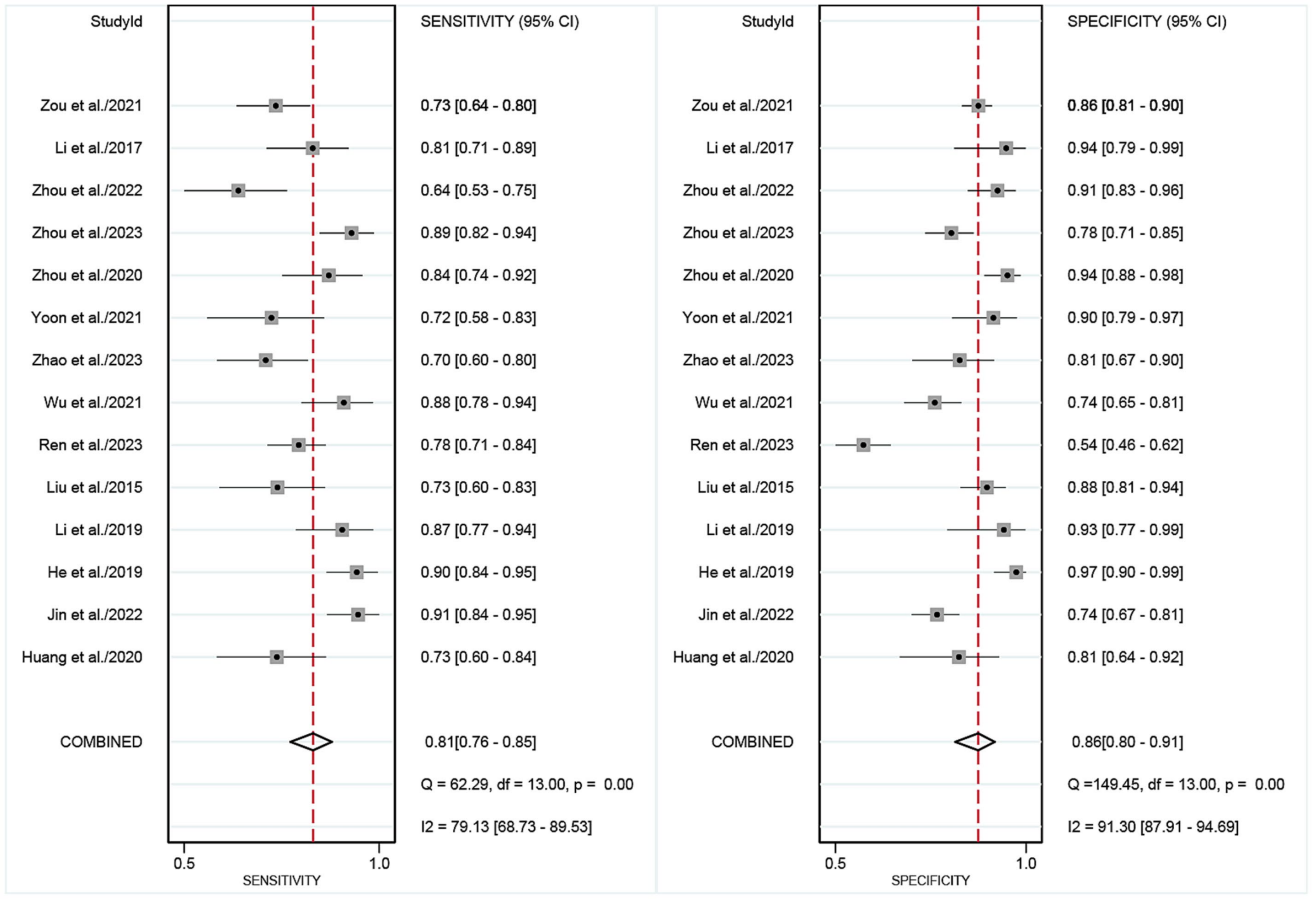
Forest plots of the combined DECT sensitivity and specificity in patients with PTC cervical lymph node metastasis. Squares denoted the sensitivity and specificity in each study, while horizontal bars indicated the 95% confidence interval. DECT, dual-energy computed tomography.

**Figure 4:**
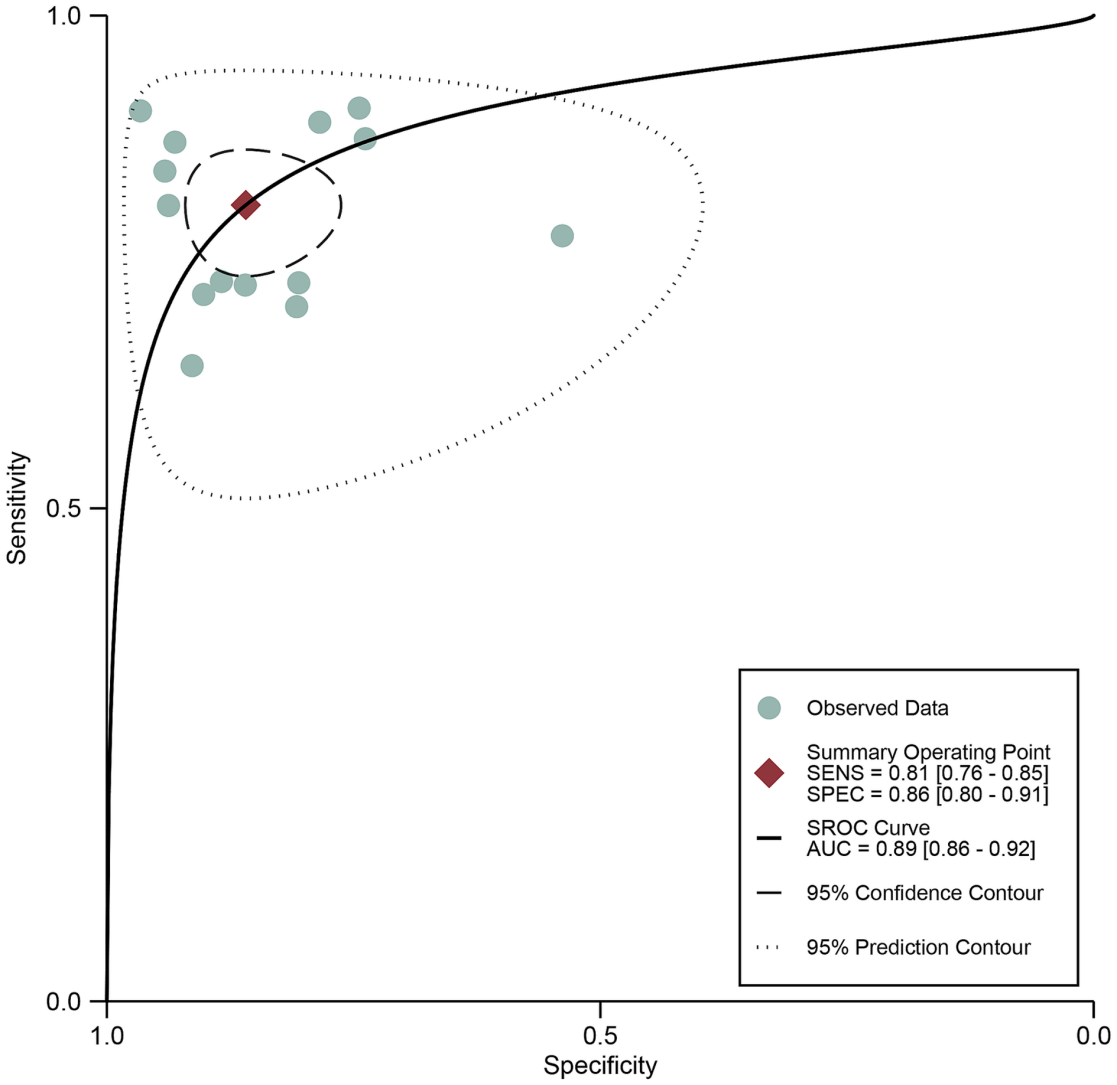
DECT summary receiver operating characteristic (SROC) curves. The summary point is the optimal combination of sensitivity and specificity. The black dotted lines surrounding each summary point indicates the 95% confidence interval. DECT, dual-energy computed tomography.

**Figure 5:**
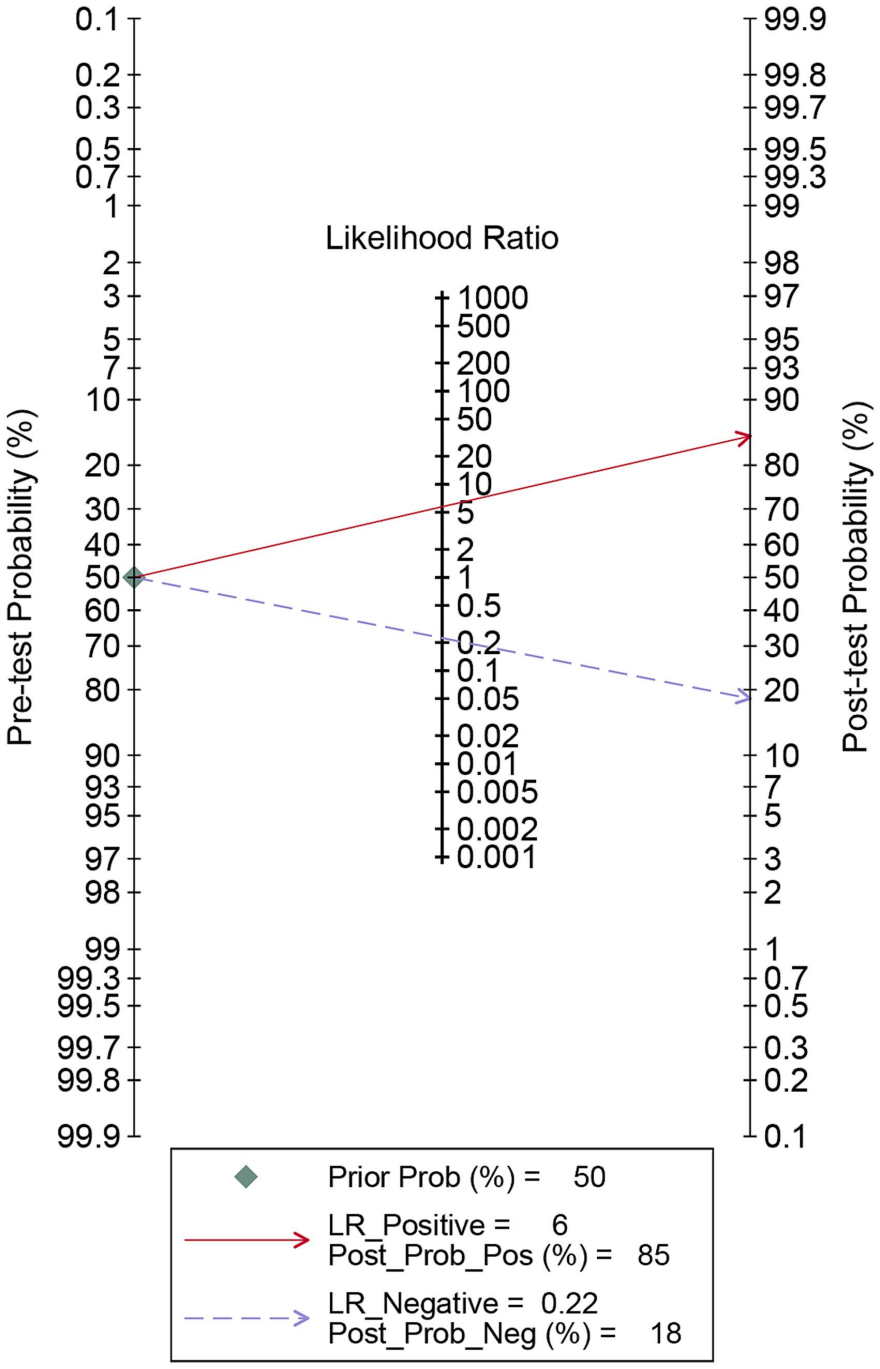
Fagan’s nomogram for DECT in assessing lymph node metastasis in papillary thyroid carcinoma. DECT, dual-energy computed tomography.

The pooled sensitivity of DECT in detecting cervical lymph node metastasis in PTC was 0.81 (95% CI, 0.76–0.85), and the specificity was 0.86 (95% CI, 0.80–0.91), as shown in Figure 3. Figure 4 presents the SROC curve for DECT, demonstrating an AUC of 0.89 (95% CI: 0.86–0.92). According to Fagan’s nomogram, for DECT, with a pre-test probability of 50%, the post-test probability was calculated as 85% for a positive result and 18% for a negative result, as illustrated in Figure 5.

Regarding the pooled sensitivity and specificity of DECT for cervical lymph node metastasis in PTC, the I^2^ values were 79.13 and 91.30%, respectively, indicating substantial heterogeneity. Meta-regression analysis for DECT revealed significant factors contributing to this heterogeneity: analysis method (*p* < 0.001 for sensitivity, *p* = 0.01 for specificity), study design (*p* < 0.001 for both sensitivity and specificity), number of patients included (*p* < 0.001 for both sensitivity and specificity), and analysis (*p* < 0.001 for both sensitivity and specificity). These variables were identified as potential causes of heterogeneity, as detailed in Table 3.

**Table 3:** Meta-regression for sensitivity and specificity for dual energy CT in detecting cervical lymph node metastases in PTC patients.

Covariate	Studies, *n*	Sensitivity (95%CI)	*p*-value	Specificity (95%CI)	*P*-value
Analysis method			<0.001		0.01
Quantitative	7	0.77 [0.70–0.84]		0.89 [0.85–0.92]	
Quantitative and visual	4	0.86 [0.80–0.93]		0.78 [0.72–0.84]	
Study design			<0.001		<0.001
Prospective	5	0.79 [0.71–0.87]		0.93 [0.88–0.97]	
Retrospective	9	0.82 [0.76–0.87]		0.81 [0.74–0.87]	
Number of patients included			<0.001		<0.001
<90	7	0.83 [0.78–0.89]		0.89 [0.82–0.95]	
≥90	7	0.78 [0.71–0.84]		0.83 [0.74–0.91]	
Analysis			<0.001		<0.001
Patient-based	5	0.73 [0.66–0.81]		0.80 [0.69–0.91]	
Lesion-based	9	0.84 [0.80–0.88]		0.88 [0.83–0.94]	

### Publication bias

**Figure 6:**
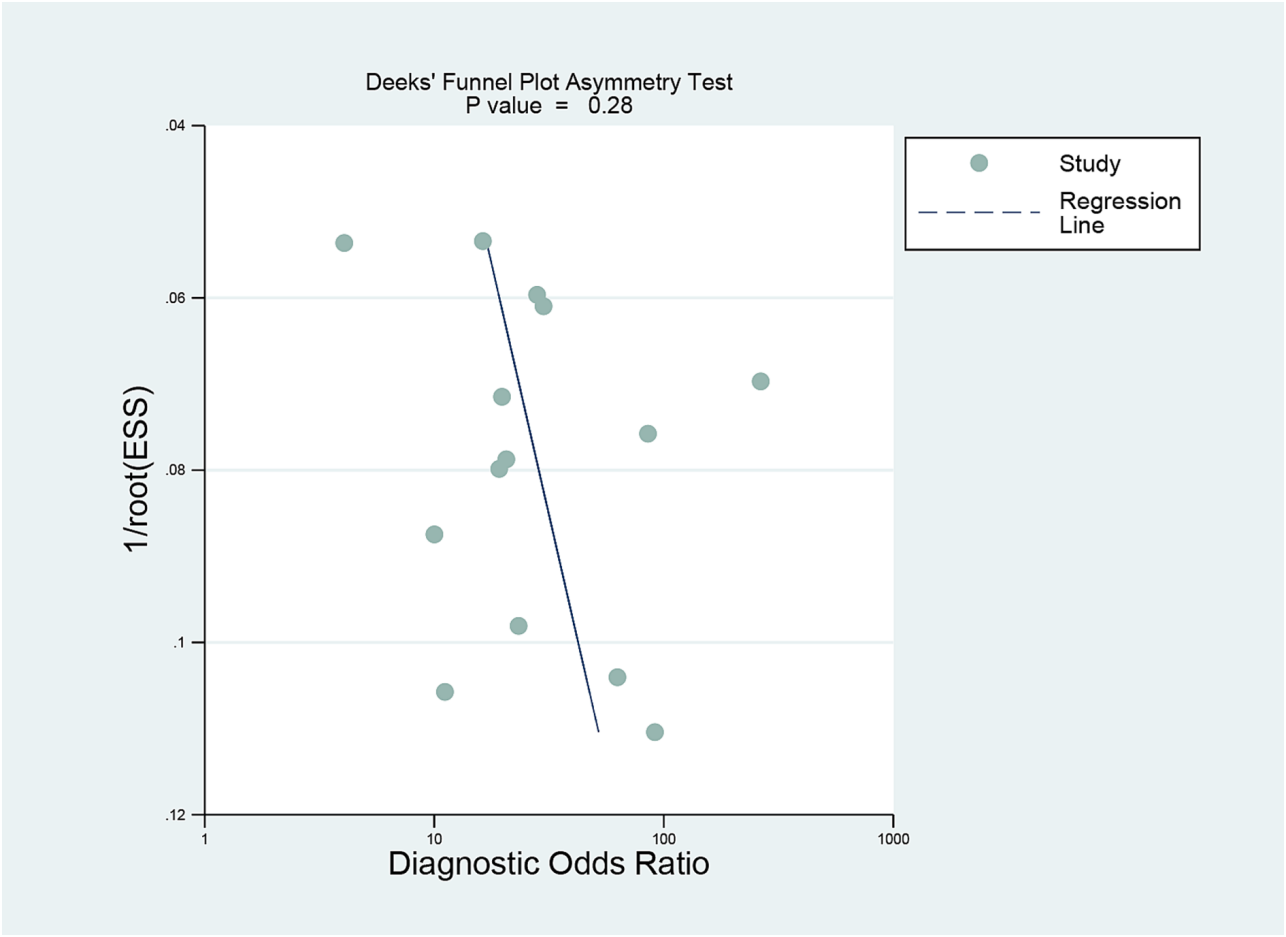
Deek’s funnel plot was used to evaluate the publication bias of DECT. *P* < 0.05 was considered significant. DECT, dual-energy computed tomography.

Deeks’ funnel plot asymmetry test showed no significant publication bias was observed for DECT (*p* = 0.28) (Figure 6).

## Discussion

This pioneering meta-analysis evaluates the efficacy of DECT in detecting cervical lymph node metastasis in PTC, amidst ongoing uncertainties and debates. A 2019 study by He et al. (28) demonstrated high diagnostic sensitivity and specificity, with the optimal DECT model exhibiting a sensitivity of 90.8% and a specificity of 80.5%. This model, integrating qualitative and quantitative factors, attained AUC values up to 0.986, emphasizing DECT’s exceptional diagnostic precision. In contrast, Zhou et al.’s (21) research did not achieve such high sensitivity and specificity levels. This divergence in findings between He et al. and Zhou et al. underscores the variable perceptions and ongoing discourse within the medical community regarding DECT’s diagnostic superiority, particularly concerning its sensitivity and specificity in detecting lymph node metastasis in PTC.

Our current meta-analysis found that the pooled sensitivity of DECT in detecting cervical lymph node metastasis in PTC was 0.81 (95% CI, 0.76–0.85), the specificity was 0.86 (95% CI, 0.80–0.91), and the AUC was 0.89 (95% CI: 0.86–0.92). These results indicate a high diagnostic accuracy of DECT. In contrast, a 2022 meta-analysis by Yang et al. (35), which included 14 studies focusing on traditional CT and US, reported a diagnostic sensitivity of 0.49 (0.44–0.54) for CT and 0.41 (0.36–0.46) for US, with a specificity of 0.91 (0.89–0.94) for CT and 0.92 (0.89–0.94) for US. Our study’s advantage lies in its focus on a more advanced imaging modality. This meta-analysis indicates that the sensitivity of traditional CT and US is inferior to our results, while the specificity is similar to our findings. The reason is that the diagnosis of lymph node metastasis using traditional CT and US primarily relies on the short axis, morphology, calcification, and cystic changes of lymph nodes (36, 37), which is highly subjective. Moreover, PTC patients are prone to metastases in small cervical lymph nodes, which often exhibit atypical morphological characteristics, thereby reducing the diagnostic utility (38). DECT can provide more comprehensive qualitative and quantitative diagnostic performance for the preoperative assessment of cervical lymph node metastasis in PTC patients, beyond the morphological signs of lymph nodes (25, 30, 39).

In our analysis, DECT’s sensitivity had an *I*^2^ of 79.13%, and specificity exhibited *I*^2^ values of 91.3%. Meta-regression identified significant factors contributing to this heterogeneity, including analysis method, study design, number of patients included, and analysis. However, the heterogeneity may also arise from differences in machine models or patient populations. Variations in DECT technology, such as differences in scanner models, imaging protocols, and post-processing software, can significantly impact the diagnostic performance (40). Different DECT systems may have varying capabilities in distinguishing between tissue types and enhancing contrast resolution, which could influence sensitivity and specificity in detecting cervical lymph node metastasis (41, 42). Additionally, patient demographic factors, such as age, gender, and ethnic background, could contribute to heterogeneity in the diagnostic accuracy of DECT. Notably, all of the studies included in our meta-analysis were conducted in Asian populations, which may lead to potential bias and limit the generalizability of our findings to other racial or regional groups. Future studies should include more diverse racial and regional patient populations to enhance the generalizability of the findings.

Our meta-analysis indicates that DECT has a superior diagnostic performance in detecting cervical lymph node metastasis in PTC, suggesting it could be a viable alternative to conventional imaging tools when only diagnostic performance is considered. The advantages of DECT include its enhanced sensitivity and ability to differentiate tissue types more accurately, which can lead to improved detection rates. However, several disadvantages must be acknowledged. The availability of DECT is limited in many medical centers due to its high cost and the need for specialized equipment (28). For hospitals, the initial investment in DECT technology involves a significantly higher purchase cost, and this expense is often passed on to patients, leading to increased medical costs for those receiving care (43). Additionally, the ongoing operational and maintenance expenses for DECT systems represent another substantial financial burden that must be considered when evaluating its feasibility for widespread use (44).

Safety concerns, while generally minimal, also need to be considered, particularly regarding the higher radiation dose associated with DECT compared to standard CT (11, 45). To mitigate these concerns, patient-specific risk assessments and the implementation of optimized imaging protocols can be employed to minimize radiation exposure without compromising diagnostic accuracy (46). The unique advantages and mechanisms of DECT differ significantly from traditional CT, and its clinical application should be tailored to the specific circumstances of each patient (27). Clinicians must weigh the benefits of DECT’s superior diagnostic capabilities against its higher costs and limited accessibility, making individualized decisions based on the clinical context and available resources. Future research should focus on identifying the most appropriate patient subgroups that would benefit the most from DECT screening in clinical practice.

Several limitations of this meta-analysis warrant consideration when interpreting the results. Firstly, the majority of the included studies are retrospective, a factor that may lead to an overestimation of the specificity and sensitivity of DECT. Accordingly, there is a need for more prospective studies to ensure accurate diagnostic performance. Secondly, the research predominantly focuses on Asian populations. To enhance the generalizability of these findings, future studies should encompass a broader spectrum of demographic groups. Additionally, the variability in DECT technology and imaging protocols across different centers could affect the generalizability of the results. Future research should aim for prospective studies with standardized DECT protocols to validate and expand upon these findings.

## Conclusion

In light of the aggregated data, this meta-analysis suggests that DECT exhibits a noteworthy diagnostic efficacy in detecting cervical lymph node metastasis in PTC patients. To substantiate these results and evaluate the practical utility of these methodologies, further prospective studies are essential.

## References

[1] Yu J Deng Y Liu T Zhou J Jia X Xiao T. Lymph node metastasis prediction of papillary thyroid carcinoma based on transfer learning radiomics. Nat Commun. (2020) 11:4807. doi: 10.1038/s41467-020-18497-3, PMID: 32968067 PMC7511309

[2] Haugen BR Alexander EK Bible KC Doherty GM Mandel SJ Nikiforov YE. 2015 American Thyroid Association management guidelines for adult patients with thyroid nodules and differentiated thyroid Cancer: the American Thyroid Association guidelines task force on thyroid nodules and differentiated thyroid Cancer. Thyroid. (2016) 26:1–133. doi: 10.1089/thy.2015.0020, PMID: 26462967 PMC4739132

[3] Shirley LA Jones NB Phay JE. The role of central neck lymph node dissection in the Management of Papillary Thyroid Cancer. Front Oncol. (2017) 7:122. doi: 10.3389/fonc.2017.00122, PMID: 28674675 PMC5474838

[4] Hoang JK Branstetter BFIV Gafton AR Lee WK Glastonbury CM. Imaging of thyroid carcinoma with CT and MRI: approaches to common scenarios. Cancer Imaging. (2013) 13:128–39. doi: 10.1102/1470-7330.2013.0013, PMID: 23545125 PMC3613791

[5] Wang H Zhao S Yao J Yu X Xu D. Factors influencing extrathyroidal extension of papillary thyroid cancer and evaluation of ultrasonography for its diagnosis: a retrospective analysis. Sci Rep. (2023) 13:18344. doi: 10.1038/s41598-023-45642-x, PMID: 37884592 PMC10603168

[6] Lang BH Shek TW Wan KY. Impact of microscopic extra-nodal extension (ENE) on locoregional recurrence following curative surgery for papillary thyroid carcinoma. J Surg Oncol. (2016) 113:526–31. doi: 10.1002/jso.24180, PMID: 26792294

[7] Genpeng L Pan Z Tao W Rixiang G Jingqiang Z Zhihui L. Prognostic implications of extranodal extension in papillary thyroid carcinomas: a propensity score matching analysis and proposal for incorporation into current tumor, lymph node, metastasis staging. Surgery. (2022) 171:368–76. doi: 10.1016/j.surg.2021.07.018 34482990

[8] Kim E Park JS Son KR Kim JH Jeon SJ Na DG. Preoperative diagnosis of cervical metastatic lymph nodes in papillary thyroid carcinoma: comparison of ultrasound, computed tomography, and combined ultrasound with computed tomography. Thyroid. (2008) 18:411–8. doi: 10.1089/thy.2007.0269, PMID: 18358074

[9] Tufano RP Noureldine SI Angelos P. Incidental thyroid nodules and thyroid cancer: considerations before determining management. JAMA Otolaryngol Head Neck Surg. (2015) 141:566–72. doi: 10.1001/jamaoto.2015.0647 25928353

[10] Cong P Wang XM Zhang YF. Comparison of artificial intelligence, elastic imaging, and the thyroid imaging reporting and data system in the differential diagnosis of suspicious nodules. Quant Imaging Med Surg. (2024) 14:711–21. doi: 10.21037/qims-23-788, PMID: 38223033 PMC10784040

[11] Liu X Ouyang D Li H Zhang R Lv Y Yang A. Papillary thyroid Cancer: Dual-energy spectral CT quantitative parameters for preoperative diagnosis of metastasis to the cervical lymph nodes. Radiology. (2015) 275:167–76. doi: 10.1148/radiol.14140481 25521777

[12] Park VY Han K Kim HJ Lee E Youk JH Kim EK. Radiomics signature for prediction of lateral lymph node metastasis in conventional papillary thyroid carcinoma. PLoS One. (2020) 15:e0227315. doi: 10.1371/journal.pone.0227315, PMID: 31940386 PMC6961896

[13] Suryavanshi S Kumar J Manchanda A Singh I Khurana N. Comparison of CECT and CT perfusion in differentiating benign from malignant neck nodes in oral cavity cancers. Eur J Radiol Open. (2021) 8:100339. doi: 10.1016/j.ejro.2021.100339, PMID: 33850970 PMC8039829

[14] Zhu Y Song Y Xu G Fan Z Ren W. Causes of misdiagnoses by thyroid fine-needle aspiration cytology (FNAC): our experience and a systematic review. Diagn Pathol. (2020) 15:1. doi: 10.1186/s13000-019-0924-z, PMID: 31900180 PMC6942345

[15] Ahmad MI Liu L Sheikh A Nicolaou S Dual-energy CT. Impact of detecting bone marrow oedema in occult trauma in the emergency. BJR Open. (2024):6. doi: 10.1093/bjro/tzae025, PMID: 39345237 PMC11427222

[16] Richtsmeier D Rodesch PA Iniewski K Bazalova-Carter M. Material decomposition with a prototype photon-counting detector CT system: expanding a stoichiometric dual-energy CT method via energy bin optimization and K-edge imaging. Phys Med Biol. (2024) 69. doi: 10.1088/1361-6560/ad25c8, PMID: 38306974

[17] Hua CH Shapira N Merchant TE Klahr P Yagil Y. Accuracy of electron density, effective atomic number, and iodine concentration determination with a dual-layer dual-energy computed tomography system. Med Phys. (2018) 45:2486–97. doi: 10.1002/mp.12903, PMID: 29624708

[18] Nagano H Takumi K Nakajo M Fukukura Y Kumagae Y Jinguji M. Dual-energy CT-derived Electron density for diagnosing metastatic mediastinal lymph nodes in non-small cell lung Cancer: comparison with conventional CT and FDG PET/CT findings. AJR Am J Roentgenol. (2022) 218:66–74. doi: 10.2214/ajr.21.26208, PMID: 34319164

[19] Wang X Liu D Zeng X Jiang S Li L Yu T. Dual-energy CT quantitative parameters for the differentiation of benign from malignant lesions and the prediction of histopathological and molecular subtypes in breast cancer. Quant Imaging Med Surg. (2021) 11:1946–57. doi: 10.21037/qims-20-825, PMID: 33936977 PMC8047348

[20] Luo S Sha Y Wu J Lin N Pan Y Zhang F. Differentiation of malignant from benign orbital tumours using dual-energy CT. Clin Radiol. (2022) 77:307–13. doi: 10.1016/j.crad.2021.12.019, PMID: 35094818

[21] Zhou Y Geng D Su G-Y Chen X-B Si Y Shen M-P. Extracellular volume fraction derived from Dual-layer spectral detector computed tomography for diagnosing cervical lymph nodes metastasis in patients with papillary thyroid Cancer: a preliminary study. Oncology. (2022) 12:12. doi: 10.3389/fonc.2022.851244, PMID: 35756662 PMC9213667

[22] Ren Y Lu S Zhang D Wang X Agyekum EA Zhang J. Dual-modal radiomics for predicting cervical lymph node metastasis in papillary thyroid carcinoma. J Xray Sci Technol. (2023) 31:1263–80. doi: 10.3233/xst-230091, PMID: 37599557

[23] Sharifabadi AD McInnes MDF Bossuyt PMM. PRISMA-DTA: an extension of PRISMA for reporting of diagnostic test accuracy systematic reviews. Clin Chem. (2018) 64:985–6. doi: 10.1373/clinchem.2018.289637, PMID: 32100836

[24] Jin D Ni X Zhang X Yin H Zhang H Xu L. Multiphase Dual-energy spectral CT-based deep learning method for the noninvasive prediction of head and neck lymph nodes metastasis in patients with papillary thyroid Cancer. Front Oncol. (2022) 12:869895. doi: 10.3389/fonc.2022.869895, PMID: 35515110 PMC9065438

[25] Wu YY Wei C Wang CB Li NY Zhang P Dong JN. Preoperative prediction of cervical nodal metastasis in papillary thyroid carcinoma: value of quantitative dual-energy CT parameters and qualitative morphologic features. Am J Roentgenol. (2021) 216:33760651:1335–43. doi: 10.2214/AJR.20.23516 33760651

[26] Huang YL Jiang YM Zhang ZH Zhao W Jiang Y Lin Y. Diagnostic value of dual-energy ct iodine for characterization of papillary thyroid micro carcinoma and better prediction of metastatic cervical lymph nodes. Iran J Radiol. (2020) 17:1–9. doi: 10.5812/iranjradiol.99924

[27] Li L Cheng SN Zhao YF Wang XY Luo DH Wang Y. Diagnostic accuracy of single-source dual-energy computed tomography and ultrasonography for detection of lateral cervical lymph node metastases of papillary thyroid carcinoma. J Thorac Dis. (2019) 11:32030219:5032–41. doi: 10.21037/jtd.2019.12.45 PMC6988027 32030219

[28] He M Lin C Yin L Lin Y Zhang S Ma M. Value of Dual-energy computed tomography for diagnosing cervical lymph node metastasis in patients with papillary thyroid Cancer. J Comput Assist Tomogr. (2019) 43:31738199:970–5. doi: 10.1097/rct.0000000000000927 31738199

[29] Zhao W Shen S Ke T Jiang J Wang Y Xie X. Clinical value of dual-energy CT for predicting occult metastasis in central neck lymph nodes of papillary thyroid carcinoma. Eur Radiol. (2023) 34:16–25. doi: 10.1007/s00330-023-10004-8, PMID: 37526667

[30] Yoon J Choi Y Jang J Shin N-Y Ahn K-J Kim B-S. Preoperative assessment of cervical lymph node metastases in patients with papillary thyroid carcinoma: incremental diagnostic value of dual-energy CT combined with ultrasound. PLoS One. (2021) 16:e0261233. doi: 10.1371/journal.pone.0261233, PMID: 34898649 PMC8668122

[31] Zhou Y Su GY Hu H Ge YQ Si Y Shen MP. Radiomics analysis of dual-energy CT-derived iodine maps for diagnosing metastatic cervical lymph nodes in patients with papillary thyroid cancer. Eur Radiol. (2020) 30:6251–62. doi: 10.1007/s00330-020-06866-x, PMID: 32500193

[32] Zou Y Zhang H Li W Guo Y Sun F Shi Y. Prediction of ipsilateral lateral cervical lymph node metastasis in papillary thyroid carcinoma: a combined dual-energy CT and thyroid function indicators study. BMC Cancer. (2021) 21:221. doi: 10.1186/s12885-021-07951-0, PMID: 33663422 PMC7934388

[33] Li L Wang Y Luo DH Zhao YF Lin M Guo W. Diagnostic value of single-source dual-energy spectral computed tomography for papillary thyroid microcarcinomas. J Xray Sci Technol. (2017) 25:793–802. doi: 10.3233/XST-16242, PMID: 28621699

[34] Zhou Y Xu YK Geng D Wang JW Chen XB Si Y. Added value of arterial enhancement fraction derived from dual-energy computed tomography for preoperative diagnosis of cervical lymph node metastasis in papillary thyroid cancer: initial results. Eur Radiol. (2023) 34:1292–301. doi: 10.1007/s00330-023-10109-0, PMID: 37589903

[35] Yang J Zhang F Qiao Y. Diagnostic accuracy of ultrasound, CT and their combination in detecting cervical lymph node metastasis in patients with papillary thyroid cancer: a systematic review and meta-analysis. BMJ Open. (2022) 12:e051568. doi: 10.1136/bmjopen-2021-051568, PMID: 35788082 PMC9255397

[36] Liu Z Zeng W Liu C Wang S Xiong Y Guo Y. Diagnostic accuracy of ultrasonographic features for lymph node metastasis in papillary thyroid microcarcinoma: a single-center retrospective study. World J Surg Oncol. (2017) 15:32. doi: 10.1186/s12957-017-1099-2, PMID: 28125992 PMC5270215

[37] Roh YH Chung SR Baek JH Choi YJ Sung TY Song DE. Validation of CT-based risk stratification system for lymph node metastasis in patients with thyroid Cancer. Korean J Radiol. (2023) 24:1028–37. doi: 10.3348/kjr.2023.0308, PMID: 37793671 PMC10550739

[38] Meler-Claramonte C Avilés-Jurado FX Vilaseca I Terra X Bragado P Fuster G. Semaphorin-3F/Neuropilin-2 transcriptional expression as a predictive biomarker of occult lymph node metastases in HNSCC. Cancers. (2022) 14. doi: 10.3390/cancers14092259, PMID: 35565388 PMC9100497

[39] Yang L Luo D Li L Zhao Y Lin M Guo W. Differentiation of malignant cervical lymphadenopathy by dual-energy CT: a preliminary analysis. Sci Rep. (2016) 6:31020. doi: 10.1038/srep31020, PMID: 27498560 PMC4976355

[40] Borges AP Antunes C Curvo-Semedo L. Pros and cons of Dual-energy CT systems: "one does not fit all". Tomography. (2023) 9:195–216. doi: 10.3390/tomography9010017, PMID: 36828369 PMC9964233

[41] Tu J Lin G Chen W Cheng F Ying H Kong C. Dual-energy computed tomography for predicting cervical lymph node metastasis in laryngeal squamous cell carcinoma. Heliyon. (2024) 10:e35528. doi: 10.1016/j.heliyon.2024.e35528, PMID: 39229502 PMC11369477

[42] Fu F He A Zhang Y Li B Wan Y. Dua-energy virtual noncontrast imaging in diagnosis of cervical metastasis lymph nodes. J Cancer Res Ther. (2015) 11:202–4. doi: 10.4103/0973-1482.168185, PMID: 26506876

[43] Godreau JP Vulasala SSR Gopireddy D Rao D Hernandez M Lall C. Introducing and building a Dual-energy CT business. Semin Ultrasound CT MR. (2022) 43:355–63. doi: 10.1053/j.sult.2022.03.005, PMID: 35738821

[44] Low YL Finkelstein E. Cost-effective analysis of Dual-energy computed tomography for the diagnosis of occult hip fractures among older adults. Value Health. (2021) 24:1754–62. doi: 10.1016/j.jval.2021.06.005, PMID: 34838273

[45] Dong J Wang X Jiang X Gao L Li F Qiu J. Low-contrast agent dose dual-energy CT monochromatic imaging in pulmonary angiography versus routine CT. J Comput Assist Tomogr. (2013) 37:618–25. doi: 10.1097/RCT.0b013e31828f5020 23863541

[46] Safari A Falahati F Mahdavi M Akbarnejad M Mohammadzadeh A. Evaluation of organ dose, effective dose and cancer risk of head and neck dual-energy computed tomography. Radiat Phys Chem. (2024) 218:111539. doi: 10.1016/j.radphyschem.2024.111539

