# Supplementary material for: The diagnostic performance of dual-energy CT imaging in cervical lymph node metastasis of papillary thyroid cancer: a meta-analysis
Source: Front Med (Lausanne). 2024 Nov 6;11:1457307. doi: 10.3389/fmed.2024.1457307 (PMC11577284; doi:10.3389/fmed.2024.1457307)
Supplement: Supplementary file 1 [file Table_1.docx]

Supplementary Table 1 Search strategy in PubMed, Embase, and Web of Science.

| Database | Search strategy |
| --- | --- |
| PubMed | (((Neoplasm, Thyroid) OR ((((((((((((((((Thyroid Neoplasm[Title/Abstract]) OR (Neoplasms, Thyroid[Title/Abstract])) OR (Thyroid Carcinoma[Title/Abstract])) OR (Carcinoma, Thyroid[Title/Abstract])) OR (Carcinomas, Thyroid[Title/Abstract])) OR (Thyroid Carcinomas[Title/Abstract])) OR (Cancer of Thyroid[Title/Abstract])) OR (Thyroid Cancers[Title/Abstract])) OR (Thyroid Cancer[Title/Abstract])) OR (Cancer, Thyroid[Title/Abstract])) OR (Cancers, Thyroid[Title/Abstract])) OR (Cancer of the Thyroid[Title/Abstract])) OR (Thyroid Adenoma[Title/Abstract])) OR (Adenoma, Thyroid[Title/Abstract])) OR (Adenomas, Thyroid[Title/Abstract])) OR (Thyroid Adenomas[Title/Abstract]))) AND (("Lymph Nodes"[Mesh]) OR (((Lymph Node[Title/Abstract]) OR (Node, Lymph[Title/Abstract])) OR (Nodes, Lymph[Title/Abstract])))) AND ((Dual source CT) OR ((((((dual-energy ct[Title/Abstract]) OR (dual-energy computed tomography[Title/Abstract])) OR (Dual Energy CT[Title/Abstract])) OR (DECT[Title/Abstract])) OR (Energy spectrum CT[Title/Abstract])) OR (Spectral CT[Title/Abstract]))) |
| Embase | ('dual-energy ct'/exp OR ‘dual-energy computed tomography’:ab,ti OR ‘Dual Energy CT’:ab,ti OR ‘DECT’:ab,ti OR ‘Energy spectrum CT’:ab,ti OR ‘DECT’:ab,ti OR ‘Spectral CT’:ab,ti OR ‘Dual source CT’:ab,ti ) AND ( 'Neoplasm, Thyroid'/exp OR ' Thyroid Neoplasm':ab,ti OR 'Neoplasms, Thyroid':ab,ti OR ' Thyroid Carcinoma':ab,ti OR 'Carcinoma, Thyroid':ab,ti OR 'Carcinomas, Thyroid':ab,ti OR 'Thyroid Carcinomas':ab,ti OR 'Cancer of Thyroid':ab,ti OR 'Thyroid Cancers':ab,ti OR 'Thyroid Cancer':ab,ti OR 'Cancer, Thyroid':ab,ti OR 'Cancers, Thyroid':ab,ti OR 'Cancer of the Thyroid':ab,ti OR 'Thyroid Adenomas':ab,ti) |
| Web of Science | TS=(“Neoplasm, Thyroid” OR “Thyroid Neoplasm” OR “Neoplasms, Thyroid” OR “Thyroid Carcinoma”OR “Carcinoma, Thyroid” OR “Carcinomas, Thyroid” OR “Thyroid Carcinomas” OR “Cancer of Thyroid” OR “Thyroid Cancers” OR “Thyroid Cancer” OR “Cancer, Thyroid”OR “Cancers, Thyroid”OR “Cancer of the Thyroid” OR “Thyroid Adenoma” OR “Adenoma, Thyroid” OR “Adenomas, Thyroid”OR “Thyroid Adenomas”) AND TS=("dual-energy ct" OR "dual-energy computed tomography" OR " Dual Energy CT" OR “DECT” OR " Spectral CT" OR " Dual Energy CT" OR " Dual source CT") |
